# Relationships of blood proinflammatory markers with psychological resilience and quality of life in civilian women with posttraumatic stress disorder

**DOI:** 10.1038/s41598-019-54508-0

**Published:** 2019-11-29

**Authors:** Risa Imai, Hiroaki Hori, Mariko Itoh, Mingming Lin, Madoka Niwa, Keiko Ino, Sei Ogawa, Atsushi Sekiguchi, Hiroshi Kunugi, Tatsuo Akechi, Toshiko Kamo, Yoshiharu Kim

**Affiliations:** 1 Department of Psychiatry and Cognitive-Behavioral Medicine, Nagoya City University Graduate School of Medical Sciences, Nagoya, Japan; 2 Department of Behavioral Medicine, National Institute of Mental Health, National Center of Neurology and Psychiatry, Tokyo, Japan; 3 Graduate School of Humanities and Social Sciences, Nagoya City University, Nagoya, Japan; 4 Department of Mental Disorder Research, National Institute of Neuroscience, National Center of Neurology and Psychiatry, Tokyo, Japan; 5 Wakamatsu-cho Mental and Skin Clinic, Tokyo, Japan

**Keywords:** Interleukins, Chronic inflammation, Prognostic markers, Post-traumatic stress disorder, Quality of life

## Abstract

Individuals with posttraumatic stress disorder (PTSD) show low resilience and impaired quality of life (QOL). Accumulating evidence shows that PTSD is associated with increased inflammation. Studies suggest that inflammation can be a key mechanism underlying low resilience/QOL, but this relationship has been understudied in individuals with PTSD. Here, we investigated the association of blood proinflammatory markers with self-reported resilience and QOL in civilian women with PTSD. Fifty-six women with PTSD and 73 healthy control women participated in this study. Resilience was assessed using the Connor-Davidson Resilience Scale. QOL was assessed using the World Health Organization Quality of Life-BREF. Blood samples were collected for the measurement of three proinflammatory markers including interleukin-6 (IL-6), high-sensitivity tumor necrosis factor-α, and high-sensitivity C-reactive protein (hsCRP). Compared to controls, patients showed significantly higher IL-6 levels and lower resilience and QOL. In patients, IL-6 levels were significantly negatively correlated with resilience, and hsCRP levels were significantly negatively correlated with psychological QOL. These results show that increased levels of proinflammatory markers including IL-6 and hsCRP are associated with lower psychological resilience and QOL in PTSD patients. Our findings suggest that interventions and treatments targeting inflammation may aid in the recovery from PTSD and lead to better prognosis.

## Introduction

Posttraumatic stress disorder (PTSD) is a debilitating psychiatric condition that can develop after a major traumatic event, often leading to a chronic course and severe functional impairment. Lifetime prevalence of PTSD is estimated at approximately 3.9% worldwide^1^. It is characterized by intrusion symptoms associated with the traumatic event, avoidance, hyperarousal, and negative alterations in cognitions and mood^2^. Besides these psychobehavioral symptoms, it has been increasingly recognized that PTSD is associated with elevated rates of somatic morbidities such as obesity, diabetes, cardiovascular disease, and early mortality^3,4^. Moreover, individuals with PTSD have poor subjective quality of life (QOL)^5^.

It is well known that individuals with PTSD show lower psychological resilience, an ability to successfully adapt to adverse conditions and recover from them^6^. According to Connor and Davidson (2003), psychological resilience refers to a range of personal qualities including hardiness, personal control, optimism and self-efficacy. As these qualities can play an integral role in recovery from trauma/PTSD, resilience is considered particularly relevant to trauma research^7^. Psychological resilience is shown to be significantly associated with QOL in various psychiatric conditions, such as major depressive disorder^8^, bipolar disorder^9^ and schizophrenia^10^. It is therefore conceivable that the low resilience will be associated with poor QOL in PTSD as well and that this low resilience/QOL can determine psychological and physical well-being of the afflicted individual.

A growing body of evidence indicates that PTSD is associated with alterations in the immune and inflammatory systems^11,12^. Epidemiological studies have reported that PTSD can increase the risk of multiple physical illnesses where immune activation plays a major role, such as metabolic syndrome^13^, autoimmune disease^14^ and atherosclerotic cardiovascular disease^15^. Previous biomarker studies including ours have shown elevated levels of proinflammatory markers such as interleukin-6 (IL-6), tumor necrosis factor-α (TNF-α) and C-reactive protein (CRP) in the blood of patients with PTSD^16–18^. Moreover, hypothesis-free genome-wide^19^, blood DNA methylome^20^ and blood transcriptome^21,22^ studies have identified genes and pathways involved in the immune/inflammatory system as the most dysregulated in patients with PTSD compared to controls.

Some studies have suggested that trauma exposure itself, irrespective of the presence/absence of PTSD diagnosis, can reduce resilience^23^, cause functional impairment^24^, and lead to elevated inflammation^25^. Still, a number of studies comparing inflammatory marker levels between PTSD patients and trauma-exposed non-PTSD controls have demonstrated that the former subjects show significantly elevated levels than the latter subjects^26–29^. This suggests that PTSD can be associated with increased inflammation beyond the possible effect of trauma exposure.

Various lines of research have demonstrated that elevated inflammation is associated with low psychological resilience^30,31^ and QOL^32,33^. Indeed, alterations in the immune/inflammatory system is considered one of the most important biological factors contributing to resilience^30,31^. For example, higher blood IL-6 levels have been reported to be associated with lower resilience, such as less self-efficacy and greater pessimism, in children^34^, in adults with a history of adverse childhood experiences^35^, and in the general population^36^. Regarding QOL, increased inflammation as indexed by higher blood CRP levels has been associated with lower self-reported QOL in schizophrenia^37^, in depression^38^, and in older adults^39^. Additionally, intervention using interferon can decrease QOL^40^.

These findings together suggest that increased inflammation may be the key mechanism by which PTSD is associated with low psychological resilience and QOL. To our knowledge, although there are two studies that examined this association in patients with PTSD related to combat exposure^41,42^, no studies have explored such an association in other populations than male military-related trauma. However, it would be of relevance to investigate this association in a female population, considering the evidence that women with PTSD have even poorer QOL than men^43^ and that the association of inflammation with fatigue^44^ and social disconnection^45^ is stronger in women than in men.

The aim of this study was to examine the association of blood proinflammatory markers, including IL-6, TNF-α and CRP, with psychological resilience/QOL assessed by well-established self-report questionnaires in civilian women with PTSD and in healthy control women. We hypothesized that elevated inflammation would be significantly associated with poor resilience/QOL in patients and that this association would be more clearly observed in patients than in controls, given the evidence for increased inflammation and poor resilience/QOL in PTSD patients compared to healthy controls.

## Results

### Sample characteristics

A total of 56 women with PTSD and 73 healthy control women participated in this study. Demographic, clinical and psychological characteristics and serum inflammatory marker levels in patients and controls are presented in Table 1. There were no significant differences between patients and controls in age, smoking status, or body mass index (BMI; calculated as weight in kilograms divided by height in meters squared). Education levels were significantly lower in patients than in controls.

**Table 1:** Demographic and clinical/psychological variables and inflammatory markers in PTSD patients and healthy controls.

Variable	PTSD patients (n = 56)	Healthy controls (n = 73)	Analysis		
			Statistic	d.f.	p
Age, years: mean ± SD	39.2 ± 9.5	35.6 ± 12.7	^c^*t* = 1.9	126.9	0.07
Education level^a^: median (25−75 percentile)	3 (2.25−4)	4 (4−5)	Mann-Whitney *U* = 1545.0		**0.01**
Smoking: yes, n (%)	8 (14.3)	7 (9.6)	χ^2^ = 0.68	1	0.41
Body mass index: mean ± SD	21.6 ± 3.4	20.7 ± 2.7	^c^*t* = 1.8	102.2	0.08
Type of index trauma					
Interpersonal violence: yes, n (%)	45 (80.4)	0 (0.0)			
Accident: yes, n (%)	4 (7.1)	0 (0.0)			
Other: yes, n (%)	7 (12.5)	0 (0.0)			
Duration of illness, less than 6 months/6 months or more: n/n	4/52	(not applicable)			
Comorbid psychiatric disorder					
Major depressive disorder: yes, n (%)	32 (57.1)	0 (0.0)			
Bipolar disorder: yes, n (%)	3 (5.4)	0 (0.0)			
Anxiety disorder: yes, n (%)	28 (50.0)	0 (0.0)			
Obsessive-compulsive disorder: yes, n (%)	6 (10.7)	0 (0.0)			
Alcohol/substance abuse or dependence: yes, n (%)	9 (16.1)	0 (0.0)			
Medication					
Antipsychotics: yes, n (%)	14 (25.0)	0 (0.0)			
Antidepressants: yes, n (%)	31 (55.4)	0 (0.0)			
Anxiolytics: yes, n (%)	30 (53.6)	0 (0.0)			
Mood stabilizers: yes, n (%)	7 (12.5)	0 (0.0)			
Hypnotics: yes, n (%)	20 (35.7)	0 (0.0)			
Impact of Event Scale-Revised^b^; total score: mean ± SD	50.9 ± 17.4	(not assessed)			
Intrusion: mean ± SD	17.8 ± 7.9	(not assessed)			
Avoidance: mean ± SD	18.6 ± 8.4	(not assessed)			
Hyperarousal: mean ± SD	14.5 ± 5.5	(not assessed)			
Posttraumatic Diagnostic Scale^b^, total score: mean ± SD	30.9 ± 9.8	(not assessed)			
AIS, total score: mean ± SD	10.6 ± 4.7	3.7 ± 3.1	^c^*t* = 9.4	89.4	<**0.001**
CD-RISC, total score: mean ± SD	41.2 ± 19.0	59.2 ± 16.5	*t* = −5.8	127	<**0.001**
WHOQOL-BREF^b^					
Physical QOL: mean ± SD	2.3 ± 0.7	3.7 ± 0.6	*t* = −12.9	126	<**0.001**
Psychological QOL: mean ± SD	2.1 ± 0.7	3.5 ± 0.6	*t* = −11.5	126	<**0.001**
IL-6 (pg/ml): median (25−75 percentile)	0.90 (0.70−1.40)	0.80 (0.60−1.00)	Mann-Whitney *U* = 2,603.0		**0.008**
hsTNF-α (pg/ml)^b^: median (25−75 percentile)	0.80 (0.60−1.00)	0.70 (0.56−0.84)	Mann-Whitney *U* = 2,329.5		0.12
hsCRP (ng/ml): median (25−75 percentile)	173.0 (82.3−443.5)	200.0 (133.5−388.5)	Mann-Whitney *U* = 1,911.5		0.53

*Abbreviations*: PTSD, posttraumatic stress disorder; d.f., degree of freedom; SD, standard deviation; AIS, Athens Insomnia Scale; CD-RISC, Connor-Davidson Resilience Scale; WHOQOL-BREF, World Health Organization Quality of Life-BREF; IL-6, interleukin-6; hsTNF-α, high-sensitivity tumor necrosis factor-α; hsCRP, high-sensitivity C-reactive protein.

*Notes*: ^a^Coded as follows: 1, junior high school graduate; 2, high school graduate; 3, some college graduate/partial university; 4, university graduate; 5, graduate school graduate.

^b^n = 55 for PTSD patients.

^c^Assumption of homogeneity of variance was not satisfied.

Bold p values represent significant results.

Most patients developed PTSD after experiencing interpersonal violence such as physical and/or sexual violence, and had been ill for more than six months. Many of the patients had psychiatric comorbidity including major depressive disorder (MDD), and were receiving psychotropic medications. In patients, the three inflammatory markers were not significantly associated with comorbid MDD or the use of any type of psychotropic drugs (all p > 0.05), except for significantly higher high-sensitivity TNF-α (hsTNF-α) levels in patients who were taking hypnotics than those who were not (Mann-Whitney U = 470.5, p = 0.034).

The mean total score on the Posttraumatic Diagnostic Scale (PDS)^46^ indicated that our PTSD patients were moderately severely ill. The indices of PTSD severity, i.e., the total score of PDS and the total score and its three subscale scores of the Impact of Event Scale-Revised (IES-R)^47^, were not significantly correlated with any of the three inflammatory marker levels in patients (all p > 0.05).

Compared to controls, patients reported significantly greater sleep problems as assessed by the Athens Insomnia Scale (AIS)^48^. The AIS scores were not significantly correlated with any of the three inflammatory markers in patients or in controls (all p > 0.05).

Patients demonstrated significantly lower resilience as assessed by the Connor-Davidson Resilience Scale (CD-RISC)^6^ and poorer physical and psychological QOL as assessed by the World Health Organization Quality of Life-BREF (WHOQOL-BREF)^49^ (all p < 0.001). Levels of education were significantly positively correlated with resilience in patients (ρ = 0.27, p = 0.045); still, the analysis of covariance (ANCOVA) with education as a covariate revealed that patients had significantly lower resilience than controls (p < 0.001).

Serum IL-6 levels were significantly higher in patients than in controls (p = 0.008), while there were no significant differences in hsTNF-α or high-sensitivity CRP (hsCRP) levels between patients and controls (Table 1).

Correlations of resilience with physical and psychological QOL were significant in the patient group (r = 0.40, p = 0.002 and r = 0.62, p < 0.001, respectively) and in the control group (r = 0.49, p < 0.001 and r = 0.70, p < 0.001, respectively).

Concerning comorbid physical illness and use of medications that can impact peripheral inflammation, three subjects (patients) had diabetes mellitus, two subjects (one patient and one control) had hypertension, two subjects (patients) had hyperlipidemia, 11 subjects (nine patients and two controls) were taking non-steroidal anti-inflammatory drugs, and one subject (patient) was taking statin.

### Association of inflammatory markers with resilience and QOL

Table 2 shows correlations of the three inflammatory markers with resilience and QOL in patients with PTSD and in healthy controls, along with the differences in correlation coefficients between groups. For this analysis, we adopted a conservative p < 0.01 to indicate statistical significance, given that 9 (i.e., 3 × 3) correlations were tested in each diagnostic group. In patients, IL-6 levels were significantly negatively correlated with resilience (p = 0.007), and hsCRP levels were significantly negatively correlated with psychological QOL (p = 0.006). In contrast, no significant correlations were observed in controls.

**Table 2:** Correlations of inflammatory markers with resilience and QOL in PTSD patients vs. healthy controls.

	PTSD patients (n = 56)			Healthy controls (n = 73)		
	Resilience (CD-RISC)	Physical QOL (WHOQOL-BREF)	Psychological QOL (WHOQOL-BREF)	Resilience (CD-RISC)	Physical QOL (WHOQOL-BREF)	Psychological QOL (WHOQOL-BREF)
IL-6	−0.36*	−0.15^a^	−0.25^a^	0.03	−0.11	0.06
hsTNF-α	−0.29^†,a^	−0.23^a^	−0.26^a^	0.16	0.07	0.22
hsCRP	−0.18	−0.16^a^	−0.37^*,a^	0.01	0.06	0.08

*Abbreviations:* PTSD, posttraumatic stress disorder; IL-6, interleukin-6; hsTNF-α, high-sensitivity tumor necrosis factor-α; hsCRP, high-sensitivity C-reactive protein; CD-RISC, Connor-Davidson Resilience Scale; WHOQOL-BREF, World Health Organization Quality of Life-BREF.

*Notes:* ^†^p < 0.05; ^*^p < 0.01.

Correlations were calculated using Spearman’s ρ.

^a^n = 55.

**Figure 1:**
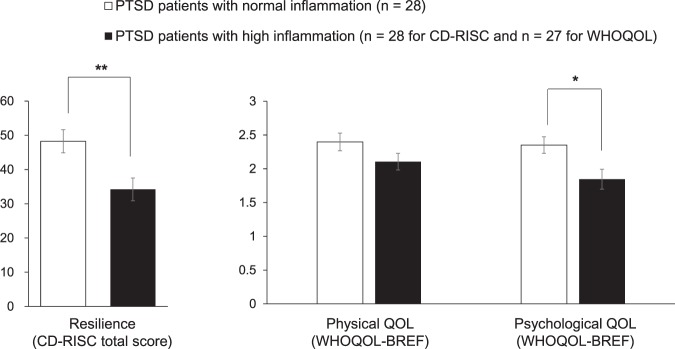
Comparison of resilience and QOL between PTSD patients with normal inflammation and those with high inflammation. Patients were split into normal (n = 28) and high (n = 28 for resilience and n = 27 for QOL) inflammation groups by the combination of 75^th^ percentile levels of three inflammatory markers in controls. Error bars indicate SEM. *p < 0.017; **p < 0.010 (by t-test). CD-RISC, Connor-Davidson Resilience Scale; WHOQOL-BREF, World Health Organization Quality of Life-BREF.

In the patient group, the three inflammatory markers were significantly correlated with each other (all ρ > 0.36, p < 0.006) and showed similar tendency regarding their correlations with resilience/QOL (Table 2). We therefore classified patients into subgroups by combining the three inflammatory markers in order to further investigate the association between inflammation and resilience/QOL in PTSD. For this purpose, patients were divided into normal and high inflammation groups based on a combination of the 75^th^ percentile levels of three inflammatory markers in the control group (i.e., IL-6: 1.0 pg/ml; hsTNF-α: 0.9 pg/ml; hsCRP: 400.0 ng/ml). More specifically, the normal inflammation group (n = 28) was defined as those patients whose levels of the three inflammatory markers were all equal to or less than the respective 75th percentile level in controls; while the high inflammation group comprised remaining 28 (27 for the QOL data) patients whose levels of one or more inflammatory marker exceeded this cutoff level. Median (25–75th percentile) levels of IL-6, hsTNF-α and hsCRP were 0.80 (0.63–0.90), 0.64 (0.50–0.80) and 138.0 (77.5–174.0) in the normal inflammation group (respectively) and 1.40 (1.10–1.73), 1.00 (0.70–1.20) and 443.0 (170.8–896.3) in the high inflammation group (respectively). For the comparison of three resilience/QOL indices between the high vs. normal inflammation groups, the Bonferroni-corrected significance threshold of p < 0.017 (i.e., p < 0.05/3) was used. As shown in Fig. 1, the high inflammation group showed significantly lower resilience (t = 3.0, d.f. = 54, p = 0.005) and psychological QOL (t = 2.6, d.f. = 53, p = 0.011) than the normal inflammation group, while no significant difference was seen for physical QOL (t = 1.6, d.f. = 53, p = 0.11). These two groups did not significantly differ in age, education levels, smoking status, comorbidity of MDD, medication (including antipsychotics, antidepressants, anxiolytics, mood stabilizers, and hypnotics), or PTSD severity (assessed by PDS and IES-R) (all p > 0.05), while, as expected, BMI was significantly higher in the high inflammation group than in the normal inflammation group (t = 2.7, d.f. = 45.7, p = 0.01). We therefore performed the ANCOVA with BMI as a covariate, which confirmed that the high inflammation group showed significantly lower resilience (F(1,53) = 8.7, p = 0.005) and psychological QOL (F(1,52) = 5.2, p = 0.026) than the normal inflammation group.

## Discussion

In this study we first confirmed our previous finding of the significantly higher serum IL-6 levels in patients with PTSD than in controls^18^, using an expanded sample. Next, as expected, resilience and QOL were found to be significantly lower in patients than in controls, and resilience and QOL were significantly positively correlated with each other within each diagnostic group. Then, our main findings can be summarized as follows. In patients, IL-6 levels were significantly negatively correlated with resilience, and hsCRP levels were significantly negatively correlated with psychological QOL. In addition, patients with high levels of inflammation for at least one proinflammatory marker showed significantly lower resilience and psychological QOL than the remaining patients without any signs of inflammation.

Acute inflammation, caused for example by bacterial infection, will itself be beneficial as it represents a part of body’s healing process; however, chronic low-grade inflammation can negatively affect health and well-being. To our knowledge, there are only a couple of studies that have examined the relationship between inflammation and resilience/QOL in individuals with PTSD. One study, focusing on health-related QOL, reported a significant association between higher plasma IL-6 levels and lower QOL in U.S. military personnel with PTSD and depression^42^. This finding obtained from a predominantly male sample is in line with the present finding from a female sample. In contrast, and somewhat unexpectedly, another study that examined psychological resilience reported a significant association of higher serum IL-6 and IFN-γ levels with *greater* resilience in male combat veterans, a majority of whom had PTSD^41^. In that study, since higher proinflammatory marker levels were also significantly associated with less symptom severity, the etiological significance of increased inflammation would be different from a majority of previous studies on inflammation in PTSD^16^; such discrepant results may be due to the heterogeneity of PTSD. Apart from PTSD, the observed association between higher CRP levels and lower QOL is in line with previous findings obtained in various populations such as patients with schizophrenia^37^ and those with depression^38^.

It should also be noted that there were no significant associations between the inflammatory markers and physical QOL in patients, in contrast to the significant association between inflammatory markers and psychological QOL. These results might raise the possibility that inflammation in PTSD is involved more in psychological rather than physical aspects of QOL. However, our findings are not in agreement with previous studies that reported significant relationships between inflammation and physical QOL^32,38,42^. This inconsistency may be ascribed to differential measures for assessing QOL between studies, given that the present study used WHOQOL-BREF whereas the previous studies used the 36-item (or 12-item) Short-Form Health Survey (SF-36/SF-12)^32,38,42^. Notably, it is pointed out that SF-36 and WHOQOL-BREF measure distinct concepts of QOL; the former will measure more objective QOL, i.e., perceived states, capabilities and functioning while the latter will measure subjective QOL, i.e., satisfaction with these states, capacities and functioning^50^. This might suggest that, compared to physical QOL, psychological QOL measured by WHOQOL-BREF would be more closely associated with resilience, and as such, may be more sensitively linked to the effect of inflammation. Supporting this, the correlation of resilience with psychological QOL was considerably stronger than that with physical QOL in both patients and controls.

In controls, none of the three inflammatory markers was significantly associated with resilience or QOL. This absence of a significant association may be because our controls consisted of psychiatrically healthy, young to middle-aged adults who collectively demonstrated high levels of resilience and QOL. Indeed, previous studies that reported significant relationships between inflammatory markers and resilience/QOL in the general population did not exclude individuals with psychiatric disorders^32,35,36,39^. Moreover, these studies targeted relatively high risk individuals (among the general population) for low resilience/QOL; for resilience, individuals with adverse childhood experiences^35^ and low-income, highly traumatized individuals^36^ were employed; for QOL, older adults were studied^32,39^.

To disentangle the effect of PTSD from that of trauma exposure itself, it would be necessary to examine a trauma-exposed control group, in addition to the PTSD patients and non-exposed healthy controls. In the present study, we were not able to include the trauma-exposed healthy control group, although we had obtained the same set of data from 18 trauma-exposed healthy subjects. However, these data were excluded from the analyses since the sample size (n = 18) was considered too small for the main correlation analysis between inflammatory markers and resilience/QOL. Still, as a reference, we only compared the three resilience/QOL indices between the three groups, i.e., PTSD patients (n = 56), trauma-exposed healthy subjects (n = 18), and non-trauma-exposed healthy subjects (n = 73), as this comparison alone may be of some help in interpreting the present results. This analysis was made by ANOVA with Bonferroni-corrected post-hoc pairwise comparisons, which revealed that the three resilience/QOL scores were significantly lower in PTSD patients than in trauma-exposed healthy controls and in non-trauma-exposed healthy controls (all p < 0.01), with no significant differences between the latter two control groups (all p > 0.2). These results suggest that the significantly lower resilience/QOL could be more strongly associated with PTSD than trauma exposure itself.

It would also be of importance to examine whether the association between inflammation and resilience/QOL varies among different inflammatory markers. In this light, our results of the significant correlations between IL-6 levels and resilience and between CRP levels and QOL could be considered as consistent with previous findings, as reviewed earlier^32,34–39^. This suggests that proinflammatory cytokines such as IL-6 may be more involved in the mechanism of psychological resilience whereas acute-phase proteins like CRP may be more associated with general well-being. Still, it is also possible that the absence of significant correlation (e.g., between IL-6 and QOL or between CRP and resilience) can be due to type II errors, considering that there was the similar tendency of correlation across significant results and non-significant ones in patients (Table 2). In addition, the comparison of patients based on the summary index of three inflammatory markers indicated that any one of these markers can be associated with lower resilience and psychological QOL. Taken together, it may be that the three proinflammatory markers examined here are generally associated with lower resilience and QOL, with some specific components of the association between IL-6 and resilience and between CRP and QOL.

Despite the significant relationship with resilience and psychological QOL, the three inflammatory marker levels were not significantly associated with symptom severity in patients. This implies that increased inflammation in PTSD would be linked more to relatively stable psychological traits (like resilience) and long-term outcomes (like QOL) than to varying symptom severity. This possibility is compatible with previous findings from longitudinal studies; for example, a study in male veterans showed that plasma CRP levels were prospectively associated with PTSD symptom emergence, indicating that inflammation could represent a risk factor for developing PTSD^51^. Consistently, an RNA-seq based transcriptome study demonstrated that dysregulated innate immune network at pre-deployment was associated with the development of PTSD post-deployment^52^. These findings including ours suggest that the increased inflammatory activity would be a lasting feature of PTSD contributing to vulnerability and long-term outcomes. On the other hand, it is reported that women in recovery from PTSD have similar levels of inflammatory markers (including IL-6 and CRP) and QOL as non-traumatized controls^53^. It should be noted that this finding does not necessarily contradict to the evidence suggesting inflammation as a preexisting vulnerability factor because it is possible that the increased inflammation that lasted from before PTSD onset can be normalized after the successful treatment of the disorder.

Although the mechanism underlying the relationships of blood inflammatory markers with psychological resilience and QOL in PTSD cannot be inferred from this study, this may be discussed based on the extant evidence. First, resilience^54^, subjective well-being^55^, and regulation of positive emotion^56^ are shown to be associated with structural and/or functional changes in specific brain regions. Next, although the present study used blood inflammatory markers, such peripheral inflammation can mirror CNS inflammation, given that circulating proinflammatory cytokines are shown to affect the brain via several mechanisms^57^. In line with this, human neuroimaging studies have demonstrated that peripheral inflammation can influence brain function^58^. It is also shown that immune mediators affect the way the brain processes information and responds to it physiologically and behaviorally, which will include the potential influences of the immune/inflammatory system on emotional and behavioral factors associated with resilience^30,57^. Furthermore, it is suggested that the gut microbiota modulates the bidirectional relationships between resilience and the immune system^30^.

There were several limitations to the present study. First, the cross-sectional nature of this study does not allow causal relationships to be determined. The causality between inflammation and psychological features in PTSD is likely complex; it is argued that both directions of causality between inflammation and resilience are possible^30^. Second, as sample size was not very large, some of the non-significant results may have actually represented type II errors (please also refer to the power calculation result below). Third, the absence of trauma-exposed healthy control group (for the main correlation analysis) precluded any definitive conclusion as to the potential effect of trauma exposure itself on the observed results in the patient group. Fourth, although there were no differences in inflammatory marker levels between patients with vs. without comorbid MDD, the potentially confounding effects of comorbid psychiatric disorders including MDD cannot be totally excluded. Finally, most of the patients were on psychotropic medications which might influence inflammation, although no significant associations were observed between any type of psychotropic drug use and inflammatory marker levels except for the significant association between hypnotic use and hsTNF-α levels.

In summary, this study shows that increased levels of proinflammatory markers including IL-6 and hsCRP are associated with lower psychological resilience and QOL in patients with PTSD. These findings suggest that interventions and treatments targeting inflammation may aid in the recovery from PTSD and lead to better prognosis. At the same time, efforts can be directed to promoting psychological resilience and well-being, which might help normalize inflammatory pathology. Future longitudinal studies that investigate the causality, or temporal relationship, between inflammation and resilience/QOL in PTSD are warranted.

## Methods

### Participants

This study was conducted at 3 institutes in Japan: National Center of Neurology and Psychiatry (NCNP) (located in Tokyo), Tokyo Women’s Medical University (Tokyo), and Nagoya City University (Aichi). The study was approved by the ethics committee of each institute, and was conducted in accordance with the Declaration of Helsinki. Written informed consent was obtained from all participants after they had received a detailed explanation of the study.

All participants recruited for this study, including both patients and controls, were Japanese women aged between 19 to 64 years. A total of 56 patients with PTSD (age range: 19–59 years) were enrolled. They were recruited at the 3 institutes and their affiliated hospitals/clinics in Tokyo and Aichi (two metropolitan areas in Japan). Most patients were outpatients at these hospitals and clinics, and their attending doctors were asked to inform the researcher of all potentially eligible patients. The remaining few patients were outpatients at the nearby clinics and were recruited though advertisements on our website. All patients had already been diagnosed as having PTSD by their attending clinicians. Experience of traumatic events and diagnosis of PTSD were confirmed using the Japanese version^59^ of the PDS^39^, a well-established self-report questionnaire for the diagnosis of PTSD. Then, the Japanese version^60^ of the Mini International Neuropsychiatric Interview^61^ was administered by an expert clinician or clinical psychologist to identify any other Axis-I disorders as well as PTSD. Exceptionally, one patient was diagnosed based only on M.I.N.I. and clinical interview because PDS was not available for this patient. Patients with comorbid schizophrenia and those with marked manic episodes of bipolar disorder were excluded from the study. In addition, non-trauma-exposed 73 healthy control women (age range: 21–64 years) were enrolled. They were recruited at NCNP through advertisements in free local magazines and on our website and by word of mouth. All healthy subjects were interviewed by a board-certified psychiatrist or a trained clinical psychologist, which included M.I.N.I and non-structured interview, and those who demonstrated current Axis-I disorders or obvious signs of past psychiatric disorders were excluded. Additionally, both patients and controls were excluded if they had serious physical illness or intellectual disability.

This study was conducted as part of an ongoing larger study. A significant subset of the present participants (89 of the total 129 participants: 69.0%) were also included in our previous study that focused on the association between inflammatory markers and cognitive function^18^; however, that study did not include data on resilience or QOL, and the main aim was different between the present study and that study.

### Psychological assessment

Psychological characteristics of participants were assessed by using the following five self-report questionnaires.

The PDS was created in accordance with the diagnostic criteria of PTSD in DSM-IV^46,62^. This scale comprises four parts that assess traumatic experiences reflecting Criteria A of the DSM-IV (Parts 1 & 2), PTSD severity during the past month reflecting Criteria B-D (Part 3), and the associated functional impairments (Part 4). The evaluation of PTSD severity in Part 3 consists of 17 items, which can be summed to yield a total score. Each item is scored on a 4-point scale of symptom frequency, with higher scores indicating greater symptom severity. The Japanese version of PDS has demonstrated good reliability and validity^59,63^. We administered Parts 1 & 2 to all participants in order to determine the presence/absence of traumatic experiences, and if present, Parts 3 & 4 were administered for the assessment of diagnosis and severity of PTSD. Based on this assessment, we included only non-trauma-exposed healthy subjects (n = 73) in the control group for the present analyses. On the other hand, there were 18 trauma-exposed healthy subjects from whom we had obtained the same set of data but were eventually excluded from the analyses. The reasons for excluding these trauma-exposed subjects were that 1) this group cannot be combined into the non-exposed group given the nature of this study and 2) the sample size (n = 18) was considered too small to comprise an independent group. There was one patient who did not complete this questionnaire, and valid PDS data were obtained from 55 patients (for Parts 1–4) as well as 73 controls (for Parts 1 & 2).

PTSD severity of the patients was also assessed using the validated Japanese version^64^ of the IES-R^47^, a 22-item self-report questionnaire measuring three core PTSD symptom clusters including intrusion, avoidance, and hyperarousal. Each item is scored on a 5-point scale of symptom intensity, with higher scores indicating greater symptom severity. There was one patient who did not complete this questionnaire, and valid IES-R data were obtained from 55 patients.

To assess insomnia symptoms, the validated Japanese version^65^ of the AIS^48^ was used. It comprises eight items scored on a 4-point scale, with higher scores indicating greater symptom severity. All participants, including patients and controls, completed this questionnaire.

Resilience was assessed using the validated Japanese version^66^ of the CD-RISC^6^. It is a 25-item self-report questionnaire scored on a 5-point scale, with higher scores indicating greater resilience. The total score of 25 items is used as the single outcome measure of CD-RISC. It is validated in various populations, including the general population and patients with psychiatric disorders. Factor analysis on the 25 items yielded five factors, including: personal competence, high standards, and tenacity; trust in one’s instincts, tolerance of negative affect, and strengthening effects of stress; the positive acceptance of change and secure relationships; control; and spiritual influences^6^. All participants completed this questionnaire.

QOL was assessed using the validated Japanese version^67^ of the WHOQOL-BREF^49^. WHOQOL-BREF, a short version of the 100-item World Health Organization Quality of Life (WHOQOL)^68^, is a 26-item self-report questionnaire. It is scored on a 5-point scale, with higher scores indicating better QOL. The 26 items are classified into four domains: physical health, psychological health, social relations and environment. In the present study we included only the first two domains, namely physical health and psychological health, in the analysis since inflammation is unlikely to directly impact social/environmental factors. There was one subject (patient) who did not complete this questionnaire, and valid WHOQOL-BREF data were obtained from 55 patients and 73 controls.

### Measurement of inflammatory markers

In this study we included three proinflammatory markers, i.e., IL-6, TNF-α, and CRP. Blood samples were collected from each participant around noon (before lunch), between 11:30 AM and 12:30 PM. Serum concentrations of IL-6, hsTNF-α, and hsCRP were measured at a clinical laboratory (SRL Inc., Tokyo, Japan). IL-6 levels were measured by chemiluminescent enzyme immunoassay, hsTNF-α levels were measured by enzyme-linked immunosorbent assay, and hsCRP levels were measured by nephelometry (SRL Inc., Tokyo, Japan). The detection limit for IL-6 was 0.3 pg/ml; none of the subjects showed IL-6 levels below this limit. The intra- and inter-assay coefficients of variation for IL-6 at 59.3, 170.8, and 523.0 pg/ml were all less than 2.6%. For hsTNF-α, the detection limit was 0.6 pg/ml (except that this limit was 0.15 pg/ml for 37 subjects who participated in this study after more sensitive reagent for hsTNF-α had become available); hsTNF-α levels of 17 subjects (13.3%) were below this limit. The intra- and inter-assay coefficients of variation for hsTNF-α at 0.73 and 6.3 pg/ml ranged between 2.9% and 6.0%. For hsCRP, the detection limit was 51 ng/ml; hsCRP levels of 11 subjects (8.5%) were below this limit. The intra- and inter-assay coefficients of variation for hsCRP at 356.0 and 2071.4 ng/ml were all less than 2.1%. Values under the detection limits were treated as 0 (pg/ml or ng/ml).

Data on the three inflammatory markers were available for all patients (n = 56) and controls (n = 73), except that hsTNF-α data were available for 55 patients and 73 controls because amount of blood collected from one subject (patient) was insufficient for measuring this marker.

### Statistical analysis

Averages are reported as “means ± SD”, or “median (25–75th percentile)” when appropriate. The t-test or Mann-Whitney *U* test was used to examine group differences. Categorical variables were compared using the χ^2^ test, or Fisher’s exact test when expected cell frequencies were less than five. Correlations were determined by Pearson’s r or Spearman’s rank order correlation (ρ). The use of parametric or nonparametric tests was based on the nature and distribution of data. For the analyses of inflammatory markers, nonparametric tests were used because these variables were not normally distributed as revealed by the Shapiro-Wilk test. The ANCOVA was used to examine group differences controlling for potential confounder(s).

Correlations of inflammatory marker levels with resilience (assessed by the CD-RISC total score) and physical/psychological QOL (WHOQOL-BREF) were determined by Spearman’s ρ in each diagnostic group, i.e., PTSD patients and healthy controls. Post-hoc power analysis using G*Power 3^69^ indicated that the patient sample (n = 56) had a power of 0.57 to detect the correlation coefficient observed between IL-6 and resilience (i.e., −0.36) at a two-tailed α = 0.01.

Statistical significance was set at two-tailed p < 0.05 unless otherwise specified. All statistical analyses were performed using the Statistical Package for the Social Sciences version 24.0 (IBM Corp., Tokyo, Japan), except that the statistical power calculation was made using G*Power 3.

## Data Availability

The data that support the findings of this study are available from the corresponding author, H.H., upon reasonable request.
